# The Association between Influenza Vaccine and Risk of Chronic Kidney Disease/Dialysis in Patients with Hypertension

**DOI:** 10.3390/vaccines11061098

**Published:** 2023-06-14

**Authors:** Wen-Rui Hao, Tsung-Lin Yang, Yu-Hsin Lai, Kuan-Jie Lin, Yu-Ann Fang, Ming-Yao Chen, Min-Huei Hsu, Chun-Chih Chiu, Tsung-Yeh Yang, Chun-Chao Chen, Ju-Chi Liu

**Affiliations:** 1 Division of Cardiology, Department of Internal Medicine, Shuang Ho Hospital, Taipei Medical University, New Taipei City 235, Taiwan; b8501043@tmu.edu.tw (W.-R.H.); 18516@s.tmu.edu.tw (Y.-A.F.); 17257@s.tmu.edu.tw (C.-C.C.); 15535@s.tmu.edu.tw (T.-Y.Y.); 2 Taipei Heart Institute, Taipei Medical University, Taipei 110, Taiwan; 151017@h.tmu.edu.tw (T.-L.Y.); 21514@s.tmu.edu.tw (K.-J.L.); 3 Division of Cardiology, Department of Internal Medicine, School of Medicine, College of Medicine, Taipei Medical University, Taipei 110, Taiwan; 4 Division of Cardiology, Department of Internal Medicine, Cardiovascular Research Center, Taipei Medical University Hospital, Taipei 110, Taiwan; 5 Division of Gastroenterology and Hepatology, Department of Internal Medicine, School of Medicine, College of Medicine, Taipei Medical University, Taipei 110, Taiwan; 09698@s.tmu.edu.tw (Y.-H.L.); u90223@tmu.edu.tw (M.-Y.C.); 6 TMU Research Center for Digestive Medicine, Taipei Medical University, Taipei 110, Taiwan; 7 Division of Gastroenterology and Hepatology, Department of Internal Medicine, Shuang Ho Hospital, New Taipei 235, Taiwan; 8 Division of Cardiovascular Surgery, Department of Surgery, Shuang Ho Hospital, Taipei Medical University, New Taipei City 235, Taiwan; 9 Graduate Institute of Data Science, College of Management, Taipei Medical University, Taipei 110, Taiwan; 701056@tmu.edu.tw; 10 Department of Neurosurgery, Shuang Ho Hospital, Taipei Medical University, New Taipei City 235, Taiwan; 11 Graduate Institute of Medical Sciences, College of Medicine, Taipei Medical University, Taipei 110, Taiwan

**Keywords:** hypertension, chronic kidney disease, dialysis, influenza vaccine

## Abstract

Backgrounds: Influenza vaccination could decrease the risk of major cardiac events in patients with hypertension. However, the vaccine’s effects on decreasing the risk of chronic kidney disease (CKD) development in such patients remain unclear. Methods: We retrospectively analysed the data of 37,117 patients with hypertension (≥55 years old) from the National Health Insurance Research Database during 1 January 2001 to 31 December 2012. After a 1:1 propensity score matching by the year of diagnosis, we divided the patients into vaccinated (*n* = 15,961) and unvaccinated groups (*n* = 21,156). Results: In vaccinated group, significantly higher prevalence of comorbidities such as diabetes, cerebrovascular disease, dyslipidemia, heart and liver disease were observed compared with unvaccinated group. After adjusting age, sex, comorbidities, medications (anti-hypertensive agents, metformin, aspirin and statin), level of urbanization and monthly incomes, significantly lower risk of CKD occurrence was observed among vaccinated patients in influenza season, non-influenza season and all season (Adjusted hazard ratio [aHR]: 0.39, 95% confidence level [C.I.]: 0.33–0.46; 0.38, 95% C.I.: 0.31–0.45; 0.38, 95% C.I.: 0.34–0.44, respectively). The risk of hemodialysis significantly decreased after vaccination (aHR: 0.40, 95% C.I.: 0.30–0.53; 0.42, 95% C.I.: 0.31–0.57; 0.41, 95% C.I.: 0.33–0.51, during influenza season, non-influenza season and all season). In sensitivity analysis, patients with different sex, elder and non-elder age, with or without comorbidities and with or without medications had significant decreased risk of CKD occurrence and underwent hemodialysis after vaccination. Moreover, the potential protective effect appeared to be dose-dependent. Conclusions: Influenza vaccination decreases the risk of CKD among patients with hypertension and also decrease the risk of receiving renal replacement therapy. Its potential protective effects are dose-dependent and persist during both influenza and noninfluenza seasons.

## 1. Introduction

Hypertension is a major common risk for coronary artery disease, stroke, heart failure, atrial fibrillation, aortic dissection, renal failure, and peripheral artery disease [1]. The presence of hypertension increases risk of renal disease and is an independent predictor of decreased glomerular filtration rate in the general population [2]. During middle and old age, blood pressure has direct and strong relationship with vascular and overall mortality [3]. The prevalence of hypertension in developing countries is increasing rapidly and the health burden of hypertension is growing worldwide [4]. The interaction between neurohormonal, renal, vascular mechanisms contributes different hemodynamic forms of hypertension. Over the nature course of hypertension, early endothelial dysfunction and increasing cardiac output usually progress into the late stage with increased peripheral vascular resistance and subsequently end organ damage with irreversible changes.

Chronic kidney disease (CKD) is a rapidly increasing public health problem and its prevalence is around 8–16% worldwide [5]. Several studies showed that CKD was associated with multiple complications and increased risk of cardiovascular disease [6]. Inflammation is the complex response of the tissue to various injuries. Inflammation can contribute to the progression of CKD by inducing cytokine release, increasing adhesion molecule production, and activating inflammatory cells [7]. Influenza viruses are one of most important causes of respiratory tract infection and cause widespread morbidity and mortality by inducing severe immunopathology of multiple organs with excessive innate immune response, resulting in 3–5 million infections and 250,000–500,000 lethal patients annually [8,9,10]. Complications of influenza include pneumonia, myositis, rhabdomyolysis, kidney injury, neurological diseases, and cardiovascular diseases [11]. Although most influenza infections are self-limited, severe complications of influenza in elderly patients can result in hospitalization or death [12]. Renal complications of influenza virus infection are uncommon but might be dangerous and lethal, reported by several studies [13,14].

Previous researches demonstrated influenza infection associated with increasing cardiovascular diseases, including acute myocardial infraction, myocarditis, cardiac arrhythmia, pericarditis, and heart failure [15,16]. In a study by Kristin, influenza vaccination in the elderly was associated with reduced risk of hospitalization for heart disease and cerebrovascular disease [17]. In a global meta-analysis of randomized clinical trials, the use of influenza vaccination significantly decreased the risk of major adverse cardiovascular events, especially in the highest-risk patients with more active coronary artery disease [18]. Although lots of studies investigated the protective effect of influenza vaccine on cardiovascular diseases and confirmed the benefit, the effect of influenza vaccine on renal complications is still unknown. In order to clarify the potential benefit of influenza vaccination on kidney diseases in a group of the elderly Taiwanese patients with hypertension with high risk of developing CKD, we conducted a population-based cohort study using reimbursement claims from Taiwan’s National Health Insurance Research Database (NHIRD).

## 2. Materials and Methods

### 2.1. Data Source

The Taiwan National Health Insurance (NHI) program was initiated in 1995 and has been providing thoroughly health care for 99% of the population of more than 23 million residents in Taiwan. The NHIRD, which was administered and held by the National Health Insurance Administration, consisted of outpatient visits, emergent department visits, hospital admissions, prescriptions, diseases, management, and treatments of all NHI enrollees. In order to protect patient privacy, data from the NHIRD that could be used to identify patients, medical institutions, and physicians, are encrypted and delinked before releasing the database to all researchers. Furthermore, all researchers using NHIRD must sign a written agreement declaring that they do not have any intention to acquire information which could potentially invade the privacy of patients or care providers. This study had been approved by the Joint Institutional Review Board of Taipei Medical University (approval No. N201804043).

### 2.2. Study Cohort

**Figure 1:**
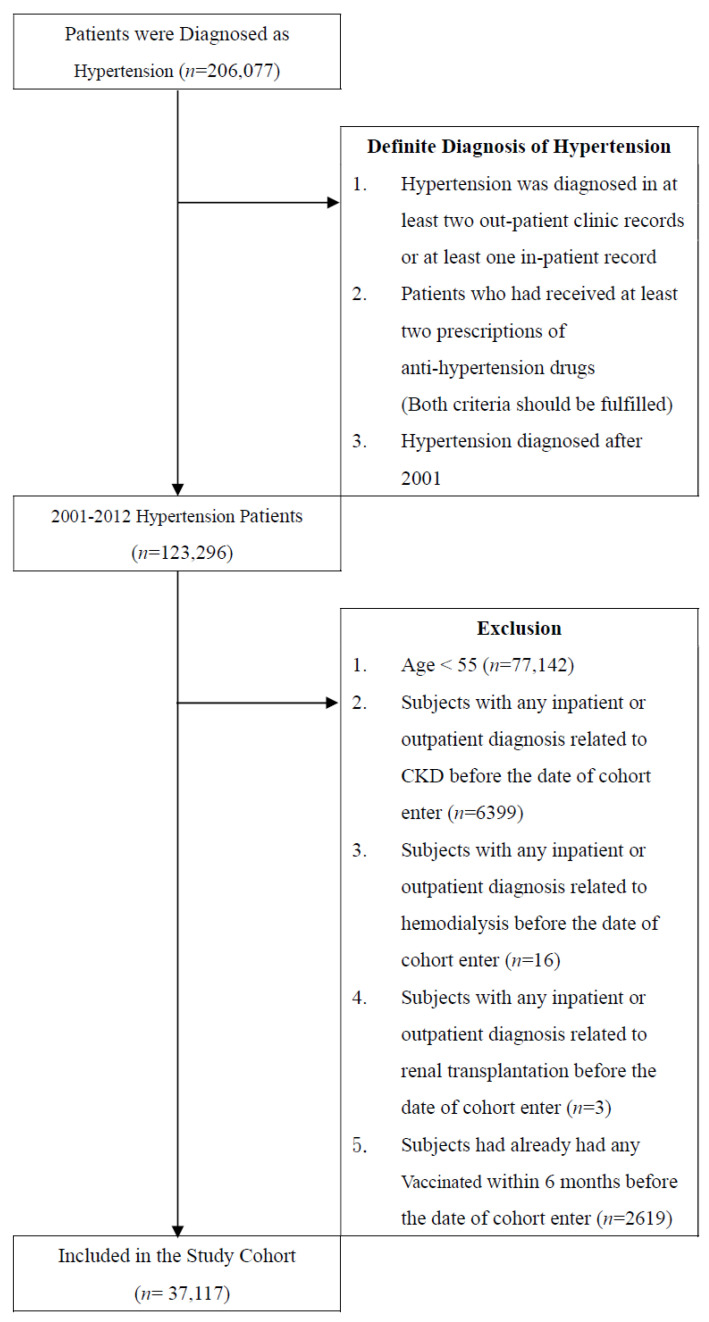
Data selection process.

This study screened all patients who were diagnosed as having hypertension based on the International Classification of Diseases, Ninth Revision, Clinical Modification (ICD-9-CM) code 401.X-405.X and who visited health-care facilities in Taiwan over 12 years from 1 January 2000, to 31 December 2012. In the first part, hypertension was diagnosed in at least two out-patient clinic records or at least one in-patient record, and patients who had received at least two prescriptions of anti-hypertension drugs. We excluded 86,179 patients due to following reasons: Patients were aged <55 years (*n* = 77,142), and patients with any inpatient or outpatient diagnosis related to CKD before the date of cohort enter (*n* = 6399), and patients with any inpatient or outpatient diagnosis related to hemodialysis before the date of cohort enter (*n* = 16), and patients with any inpatient or outpatient diagnosis related to renal transplantation before the date of cohort enter (*n* = 3), and patients had already had any Vaccinated within 6 months before the date of cohort enter (*n* = 2619). Finally Included in the Study Cohort (*n* = 37,117; Figure 1). In addition, a 1-year washout period (2000) was included to ensure that all patients in this cohort had no CKD or dialysis before enrollment. The influenza vaccines were all given by intramuscular injection. The vaccination status was recognized based on the presence of code V048 or the use of the vaccine [confirmed by drug codes of Vaxigrip^®^ (inactivated trivalent influenza vaccine, Sanofi Pasteur, France); AdimFlu S^®^ (inactivated trivalent influenza vaccine, Adimmune Corporation, Taiwan), Fluvirin^®^ (inactivated trivalent influenza vaccine, Novartis, UK)]. The primary endpoints of our study were the incidence of CKD (ICD-9-CM code 585.X) and the requirement of dialysis (NHI procedure codes) in patients with hypertension. All cohorts were followed up until the date of the diagnosis of CKD, dialysis, death, disenrollment from the NHI, or the end of 2012.

### 2.3. Potential Confounders

The following diagnoses were recorded to establish the baseline comorbidity history for each participant: diabetes; cerebrovascular diseases; dyslipidemia; heart diseases; hepatitis B virus; hepatitis C virus; cirrhosis; moderate and severe liver disease; asthma; and prescriptions for medications that include statin, metformin, aspirin, and prescriptions for anti-hypertension medications included diuretics, beta-blockers, calcium channel blocker, renin angiotensin aldosterone system (RAA) blocker. The cohort was also classified based on sociodemographic characteristics: age (categorized into 3 groups: 55–64, 65–74, and ≥75 years old), gender (male, female), Level of Urbanization (urban, suburban, and rural area), and income on monthly basis (0, 1–20,100, 20,100–30,301, and ≥30,301 in New Taiwan Dollar (NT$)).

### 2.4. Statistical Analysis

A propensity score (PS) is used to reduce selection bias and estimate the effect of vaccination by accounting for covariates that predict receiving the intervention (vaccine) by using a logistic regression model. Covariates in the main model were adjusted for PSs for age, sex, diabetes, dyslipidemia cerebrovascular diseases, heart diseases, hepatitis B virus, hepatitis C virus, cirrhosis, moderate and severe liver disease, asthma, anti-hypertension medications, statin, metformin, aspirin, level of urbanization, monthly income (Table 1). Categorical variables were compared using the chi-square test to determine the significance of differences between the vaccinated and unvaccinated groups in terms of the relationship among characteristics listed in Table 1. The unvaccinated group served as the reference arm. The hazard ratio (HR) and 95% confidence interval (CI) for the association between influenza vaccination and the risks of CKD and dialysis in patients with Hypertension were calculated using Cox proportional hazards regression. To examine the dose effect of influenza vaccination on the incidence of CKD and dialysis, we categorized patients into four groups by vaccination status: unvaccinated and those receiving 1, 2–3, and ≥4 vaccination, respectively. These data were stratified according to patients’ age, sex, comorbidity, and associated medication use. Sensitivity analysis was performed to evaluate the difference and consistency between the use of influenza vaccination and the risks of CKD and dialysis in patients with Hypertension. All statistical analyses were performed using SPSS 22.0 and SAS 9.4 software. A *p* value of <0.05 indicated statistical significance.

## 3. Results

### 3.1. Baseline Characteristics of Study Population

Total 37,117 patients with hypertension were investigated. Table 1 showed the baseline characteristics of the whole cohort, unvaccinated group and vaccinated group. In vaccinated group, there were more patients with diabetes (43.54% vs. 37.89%, *p* < 0.001), cerebrovascular disease (39.80% vs. 27.82%, *p* < 0.001), dyslipidemia (53.52% vs. 49.17%, *p* < 0.001), heart diseases (68.20% vs. 52.99%, *p* < 0.001), hepatitis C infection (4.46% vs. 3.97%, *p* = 0.002) and asthma (29.19% vs. 19.32%, *p* < 0.001). In the past medication history, vaccinated group had greater prescription of all class of antihypertension agents, long term (>365 days) statin, metformin and aspirin usage.

### 3.2. Risk of Developing Chronic Kidney Disease

After adjusting the age, sex, diabetes, dyslipidemia cerebrovascular diseases, heart diseases, hepatitis B virus, hepatitis C virus, cirrhosis, liver disease, asthma, all class of antihypertensive agents, statin, metformin, aspirin, level of urbanization and monthly income by propensity score matching, vaccinated group had significantly lower risk of developing CKD than unvaccinated group in influenza season, non-influenza season and all season (aHR: 0.39 [95% confidence interval [CI]: 0.33–0.46], 0.38 [95% CI: 0.31–0.45], 0.38 [95% CI: 0.34–0.44], respectively). Patients with age either <65 year-old or >65 year-old had significant lower risk of developing CKD in influenza season, non-influenza season and all season. Both male and female patients had significantly lower risk of CKD after receiving vaccination in influenza season, non-influenza season and all season (Table 2).

### 3.3. Risk of Receiving Hemodialysis

Among the vaccinated patients, risk of receiving hemodialysis was significantly lower than unvaccinated patients in influenza season, non-influenza season and all season (aHR: 0.40 [95% CI: 0.30–0.53], 0.42 [95% CI: 0.31–0.57], 0.41 [95% CI: 0.33–0.51], respectively). In patients with age <65 year-old or >65 year-old, the risk of receiving hemodialysis significantly reduced after vaccination in influenza season, non-influenza season and all season. Both female and male patients had reduced risk of receiving hemodialysis after vaccination in influenza season, non-influenza season and all season (Table 3).

### 3.4. Sensitivity Analysis of Vaccination in CKD Occurrence

In influenza season, with more times of vaccination, the risk of developing CKD significantly decreased after adjusting the age, sex, comorbidities, medications, level of urbanization and monthly income (aHR: 0.59 [95% CI: 0.47–0.74], 0.49 [95% CI: 0.39–0.60], 0.13 [95% CI: 0.09–0.19] in 1, 2–3 and ≥4 times of vaccination respectively) (Table 4). Subgroup analysis showed either patients with age < 65 or ≥65 year-old, the risk of CKD had significantly reduced after receiving more times of vaccination (aHR: 0.56 [95% CI: 0.41–0.76], 0.34 [95% CI: 0.23–0.50], 0.25 [0.14–0.42] in patients with age < 65 year-old, receiving 1, 2–3 and ≥4 times of vaccination respectively; 0.64 [95% CI:0.53–0.78], 0.44 [95% CI: 0.37–0.53], 0.13 [0.10–0.17] in patients with age ≥65 year-old, receiving 1, 2–3 and ≥4 times of vaccination respectively). Both female and male patients had significantly greater reduction of risk of developing CKD after receiving more times of vaccination (aHR: 0.65 [95% CI:0.51–0.84], 0.31 [95% CI: 0.23–0.42], 0.14 [0.10–0.21] in female patients, receiving 1, 2–3 and ≥4 times of vaccination respectively; 0.61 [95% CI: 0.49–0.75], 0.50 [95% CI: 0.41–0.62], 0.15 [0.11–0.21] in male patients, receiving 1, 2–3 and ≥4 times of vaccination respectively). In patients without diabetes, the risk of CKD decreased after receiving more than 2 times of vaccination (aHR: 0.74 [95% CI: 0.54–1.00], 0.59 [95% CI: 0.44–0.80], 0.14 [0.08–0.25], receiving 1, 2–3 and ≥4 times of vaccination respectively). Patients with diabetes had decreased risk of CKD after receiving more than one time of vaccination and the risk had significantly decreased by more times of vaccination (aHR: 0.47 [95% CI: 0.36–0.66], 0.0.40 [95% CI: 0.29–0.55], 0.12 [0.07–0.19], receiving 1, 2–3 and ≥4 times of vaccination respectively). Patients with prescriptions of all types of antihypertensive medications had lower risk of CKD by receiving one time of vaccination and had more significant decreasing risk of CKD than 4 times of vaccination (aHR: 0.58 [95% CI: 0.34–0.96], 0.65 [95% CI: 0.42–1.01], 0.18 [0.09–0.34], receiving 1, 2–3 and ≥4 times of vaccination respectively). Further subgroup analysis showed all patients who had prescription of diuretic, betablocker, calcium channel blocker and renin-angiotensin antagonist had decreased risk of CKD by receiving more times of vaccination. The patients who did not have the abovementioned antihypertensive medications also had significant decreasing risk of CKD. Patients with duration of statin usage history less than 28 days had significant decreased risk of CKD by receiving 1, 2–3 times and more than 4 times of vaccination (aHR: 0.61 [95% CI: 0.47–0.78], 0.46 [95% CI: 0.36–0.59], 0.12 [0.08–0.19], respectively). Patients with duration of metformin less than 28 days, 28–365 days and more than 365 days had lower risk of CKD by receiving more times of vaccination. Patients with duration of aspirin usage history less than 28 days had significant decreased risk of CKD by receiving 1, 2–3 times and more than 4 times of vaccination (aHR: 0.57 [95% CI: 0.43–0.77], 0.47 [95% CI: 0.35–0.63], 0.11 [0.07–0.19], respectively). In non-influenza season and all seasons, significant trend of lowering risk of CKD by receiving more times of vaccination was observed (aHR: 0.66 [95% CI: 0.53–0.83], 0.36 [95% CI: 0.28–0.47], 0.17 [0.12–0.24]; aHR: 0.63 [95% CI: 0.53–0.74], 0.43 [95% CI: 0.36–0.50], 0.15 [0.12–0.19], receiving 1, 2–3 and ≥4 times of vaccination, in non-influenza season and all season respectively) (Table 5 and Table 6).

### 3.5. Sensitivity Analysis of Vaccination in Undergoing Hemodialysis

Table 7 showed the reduction of risk of receiving hemodialysis in patients during influenza season. By receiving more times of vaccination, the risk of receiving hemodialysis decreased after adjusting age, sex, comorbidities and medications (aHR: 0.63 [95% CI: 0.44–0.92], 0.43 [95% CI: 0.29–0.63], 0.21 [95% CI: 0.13–0.35], receiving 1, 2–3 and ≥4 times of vaccination respectively). Patients in age less than 65 years old had reduction of risk of receiving hemodialysis after receiving more than 2 times of vaccination (aHR: 0.90 [95% CI: 0.54–0.1.50], 0.15 [95% CI: 0.05–0.48], 0.35 [95% CI: 0.14–0.86], receiving 1, 2–3 and ≥4 times of vaccination respectively). In patients elder than 65 years old, the risk of hemodialysis decreased after 1 time of vaccination (aHR: 0.47 [95% CI: 0.28–0.81], 0.50 [95% CI: 0.32–0.78], 0.17 [95% CI: 0.09–0.31], receiving 1, 2–3 and ≥4 times of vaccination respectively). Female patients had reduced risk of undergoing hemodialysis after 1 time of vaccination (aHR: 0.52 [95% CI: 0.30–0.92], 0.27 [95% CI: 0.14–0.52], 0.15 [95% CI: 0.07–0.34], receiving 1, 2–3 and ≥4 times of vaccination respectively). Male patients had reduced risk of undergoing hemodialysis after receiving more than 2 times of vaccination (aHR: 0.76 [95% CI: 0.46–1.25], 0.60 [95% CI: 0.37–1.00], 0.29 [95% CI: 0.15–0.55], receiving 1, 2–3 and ≥4 times of vaccination respectively). Patients with and without diabetes had reduced risk of receiving hemodialysis after more than 2 times of vaccination (aHR: 0.68 [95% CI: 0.43–1.07], 0.50 [95% CI: 0.31–0.80], 0.18 [95% CI: 0.09–0.36]; aHR: 0.55 [95% CI: 0.29–1.06], 0.32 [95% CI: 0.15–0.65], 0.28 [95% CI: 0.13–0.58], receiving 1, 2–3 and ≥4 times of vaccination among patient with and without diabetes respectively). Patients with and without heart disease had reduced risk of receiving hemodialysis after more than 2 times of vaccination (aHR: 0.70 [95% CI: 0.45–1.08], 0.46 [95% CI: 0.29–0.73], 0.17 [95% CI: 0.09–0.33]; aHR: 0.51 [95% CI: 0.25–1.04], 0.33 [95% CI: 0.15–0.74], 0.33 [95% CI: 0.15–0.74], receiving 1, 2–3 and ≥4 times of vaccination among patient with and without heart disease respectively). Patients with cerebrovascular disease had reduced risk of receiving hemodialysis after more than 4 times of vaccination (aHR: 0.86 [95% CI: 0.48–1.52], 0.67 [95% CI: 0.39–1.18], 0.28 [95% CI: 0.13–0.61], receiving 1, 2–3 and ≥4 times of respectively). Patients without cerebrovascular disease had reduced risk of receiving hemodialysis after 1 time of vaccination (aHR: 0.54 [95% CI: 0.33–0.88], 0.30 [95% CI: 0.17–0.53], 0.18 [95% CI: 0.09–0.36], receiving 1, 2–3 and ≥4 times of respectively). In patients who took antihypertensive agents for less than 28 days, the risk of receiving hemodialysis decreased after more than 2 times of vaccination (aHR: 0.72 [95% CI: 0.47–1.09], 0.43 [95% CI: 0.27–0.68], 0.21 [95% CI: 0.12–0.40], receiving 1, 2–3 and ≥4 times of respectively). Patents who took antihypertensive agents more than 28 days had decreased risk of receiving hemodialysis after 1 time of vaccination (aHR: 0.43 [95% CI: 0.19–0.98], 0.42 [95% CI: 0.0–0.88], 0.21 [95% CI: 0.09–0.52], receiving 1, 2–3 and ≥4 times of respectively). Patients who took either diuretic agent, beta-blocker, calcium channel blocker or RAA for more than 28 days all had decreased risk of receiving hemodialysis after more than 2 times of vaccination. Patients who took statin either for less than 28 days, 28–365 days and more than 365 days had decreased risk of hemodialysis after more than 2 times of vaccination. Patients who took metformin less than 28 days had reduced risk of hemodialysis after 1 time of vaccination (aHR: 0.57 [95% CI: 0.35–0.94], 0.39 [95% CI: 0.23–0.65], 0.25 [95% CI: 0.14–0.45], receiving 1, 2–3 and ≥4 times of respectively). In patients who took metformin more than 365 days, the risk of receiving hemodialysis reduced after more than 4 times of vaccination (aHR: 0.88 [95% CI: 0.45–1.73], 0.61 [95% CI: 0.30–1.23], 0.23 [95% CI: 0.09–0.62], receiving 1, 2–3 and ≥4 times of respectively). Patients who took aspirin for less than 28 days had reduced risk of receiving hemodialysis after 1 time of vaccination (aHR: 0.45 [95% CI: 0.25–0.79], 0.50 [95% CI: 0.30–0.82], 0.18 [95% CI: 0.09–0.38], receiving 1, 2–3 and ≥4 times of respectively). Patients took aspirin for more than 365 days had no reduction of hemodialysis risk after taking 1, 2–3 or more than 4 times of vaccination (aHR: 0.70 [95% CI: 0.28–1.77], 0.38 [95% CI: 0.14–1.05], 0.41 [95% CI: 0.17–1.03] respectively). In non-influenza season, significant reduced risk of receiving hemodialysis was observed after taking more than 2 times of vaccination (aHR: 0.90 [95% CI: 0.63–1.28], 0.42 [95% CI: 0.27–0.64], 0.12 [95% CI: 0.06–0.24], receiving 1, 2–3 and ≥4 times of respectively) (Table 8). In all season, risk of receiving hemodialysis significantly reduced after taking more than 1 times of vaccination (aHR: 0.75 [95% CI: 0.58–0.98], 0.42 [95% CI: 0.32–0.56], 0.17 [95% CI: 0.11–0.26], receiving 1, 2–3 and ≥4 times of respectively) (Table 9).

## 4. Discussion

In this nationwide population based observational study, there are several main findings. First, we found that patients with hypertension, without any history of kidney diseases, hemodialysis, or having received renal transplantation, who had received influenza vaccination, had a lower rate to develop CKD in the future. Second, the risk of receiving dialysis therapy among patients with hypertension significantly decreased after vaccination. Third, the potential renal protective effect of influenza vaccine was observed in both males and females, patients with or without chronic comorbidities and with or without medications usage. Fourth, the risk reduction of CKD occurrence or receiving dialysis decreased after influenza vaccination appeared to be dose-dependent. Fifth, the potential renal protective effect was observed during influenza season, non-influenza season and all seasons.

Previous epidemiological researches established that blood pressure was related to CKD and proteinuria [19,20]. In Hanratty’s retrospective cohort study for a medium of 3.67-year follow-up, 12.1% of 43,305 hypertensive patients developed CKD and systolic blood pressure was found associated with incident CKD [21]. Hypertension has been an important risk factor for CKD and responsible for 27% of all end-stage renal disease(ESRD) patients in America [22]. Besides, infections, such as urinary tract infection, are also known associated with renal cell damage and scarring, leading to CKD and ESRD [23]. Sepsis often results in multiple organ dysfunction and acute kidney injury, occurring in about 19% patients with moderate sepsis, 23% with severe sepsis, and 51% with septic shock [24]. Certain studies demonstrated that inflammation played a pivotal role in kidney function decline and the pathogenesis of CKD [25,26,27,28,29]. Hypertension and infection are major risk factors contributing to CKD.

In the first case report by Myking, influenza virus infection was shown associated with severe renal failure [30]. Since then, although renal complications of influenza were uncommon, small series of influenza virus infection complicated by renal failure were still reported constantly. The renal complications of influenza infection include acute kidney injury (AKI), rhabdomyolysis, hemolytic uremic syndrome, acute glomerulonephritis, disseminated intravascular coagulation, Goodpasture’s syndrome, and tubulointerstitial nephritis. In Watanabe et al.’s study, in 45 hospitalized children with influenza A virus infection, 24.4% of the patients had renal involvement and sepsis with multiple organ failure [31]. According to certain studies, approximately one third of patients with influenza virus infection during hospitalization developed AKI and some even required renal replacement therapy [32,33,34].

There are several possible mechanisms contributing development of kidney injury in patients with influenza infection. Soto-Abraham et al. reported one of five patients who died of influenza infection had acute tubular necrosis (ATN) [35]. Another study demonstrated 21 patients who died of influenza infection all exhibited mild to moderate severity of ATN and four patients had myoglobin pigment deposited in the renal tubules [36]. Carmona et al. analyzed the autopsy findings of five patients who died of influenza infection and found ATN existed in all patients without evidence of direct virus-induced kidney injury [37]. These researches implicate ATN often complicate patients with influenza infection and leading to kidney injury. Rhabdomyolysis is a lethal-threatening syndrome characterized by the release of muscle contents, including electrolytes, myoglobin, enzymes, and other sarcoplasmic proteins into the circulatory system. Acute kidney injury is a quite common complication that developed in 13% to approximately 50% patients with rhabdomyolysis [38,39]. Several studies had indicated that influenza virus infection was associated with rhabdomyolysis and led to kidney injury [40,41]. There were also some studies showing that influenza virus could be detected in urine and implicated that influenza virus would directly invade the urinary system and lead to kidney injury [42,43]. In addition, influenza infection would evoke cell mediated immunity with secretion of Th17 and Th1 cytokines. Dysregulation of cytokine expression due to viral antigen deposition in the kidney results in T-cell mediated kidney injury was found in patients with influenza virus infection [44].

There are several limitations of the present study. First, this study retrospectively analyzed population data retrospectively. Risk factors that contribute to increasing risk of CKD or dialysis were adjusted using the propensity score method to minimize the potential bias. However, future prospective studies are warranted to validate the results of present study. Second, based on the limitation of NHIRD, confounding variables such as body mass index, laboratory data such as estimated glomerular filtration rate, proteinuria and creatinine level, and smoking or alcohol consumption status could not be collected. Third, the effectiveness of the influenza vaccine could be affected by the mutation of the influenza virus in different years. In the present study, the potential renoprotective effect was also observed during the non-influenza season. However, future studies aim to compare the different vaccine effectiveness and the risk of CKD or receiving hemodialysis is warranted.

## 5. Conclusions

The risk of CKD potentially decreased after receiving influenza vaccination among patients with CKD. Moreover, the risk of dialysis also significantly decreased after influenza vaccination. The potential renoprotective effects of influenza vaccination was observed in all seasons and appeared to be dose-dependent.

## Figures and Tables

**Table 1:** Characteristic of the Sample Population.

	Whole Cohort (*n* = 37,117)		Unvaccinated (*n* = 21,156)		Vaccinated (*n* = 15,961)		*p*
	*n*	%	*n*	%	*n*	%	
Age, years (Mean ± SD)	66.41 (8.10)		64.05 (8.09)		69.55 (6.98)		<0.001
55–64	18,475	49.78	13,821	65.33	4654	29.16	<0.001
65–74	12,665	34.12	4793	22.66	7872	49.32	
≥75	5977	16.10	2542	12.02	3435	21.52	
Gender							
Female	18,324	49.37	10,251	48.45	8073	50.58	<0.001
Male	18,793	50.63	10,905	51.55	7888	49.42	
Comorbidities							
Diabetes	14,967	40.32	8017	37.89	6950	43.54	<0.001
Cerebrovascular diseases	12,239	32.97	5886	27.82	6353	39.80	<0.001
Dyslipidemia	18,946	51.04	10,403	49.17	8543	53.52	<0.001
Heart diseases	22,095	59.53	11,210	52.99	10,885	68.20	<0.001
Hepatitis B virus	692	1.86	409	1.93	283	1.77	0.259
Hepatitis C virus	1454	3.92	742	3.51	712	4.46	<0.001
Cirrhosis	1575	4.24	839	3.97	736	4.61	0.002
Moderate and Severe liver disease	485	1.31	279	1.32	206	1.29	0.813
Asthma	8746	23.56	4087	19.32	4659	29.19	<0.001
Anti-hypertension medications							
Antihypertensives	7173	19.33	3244	15.33	3929	24.62	<0.001
Diuretics	18,986	51.15	9599	45.37	9387	58.81	<0.001
Beta blocking agents	19,245	51.85	10,290	48.64	8955	56.11	<0.001
Calcium channel blocker	26,333	70.95	14,273	67.47	12,060	75.56	<0.001
RAA	22,342	60.19	11,904	56.27	10,438	65.40	<0.001
Co-medications							
Statin drugs							
<28 days	27,412	73.85	15,947	75.38	11,465	71.83	<0.001
28–365 days	6074	16.36	3416	16.15	2658	16.65	
>365 days	3631	9.78	1793	8.48	1838	11.52	
Metformin drug							
<28 days	29,703	80.03	17,106	80.86	12,597	78.92	<0.001
28–365 days	2818	7.59	1715	8.11	1103	6.91	
>365 days	4596	12.38	2335	11.04	2261	14.17	
Aspirin drug							
<28 days	21,827	58.81	13,648	64.51	8179	51.24	<0.001
28–365 days	8350	22.50	4422	20.90	3928	24.61	
>365 days	6940	18.70	3086	14.59	3854	24.15	
Level of Urbanization							
Urban	25,288	68.13	15,385	72.72	9903	62.04	<0.001
Suburban	7908	21.31	4070	19.24	3838	24.05	
Rural	3921	10.56	1701	8.04	2220	13.91	
Monthly income (NT$)							
0	4331	11.67	2125	10.04	2206	13.82	<0.001
1–20,100	12,010	32.36	6302	29.79	5708	35.76	
20,100–30,300	13,582	36.59	7335	34.67	6247	39.14	
≥30,301	7194	19.38	5394	25.50	1800	11.28	

**Table 2:** Risk of CKD among Unvaccinated and Vaccinated in Study Cohort.

All Group (*n* = 37,117)	Unvaccinated (Total Follow-Up 88,819.1 Person-Years)			Vaccinated (Total Follow-Up 97,040.0 Person-Years)			Adjusted HR † (95% C.I.)
	No. of Patients with Cancer	Incidence Rate (per 10^5^ Person-Years) (95% C.I.)		No. of Patients with Cancer	Incidence Rate (per 10^5^ Person-Years) (95% C.I.)		
Whole cohort							
Influenza season	451	507.8	(460.9, 554.6)	233	240.1	(209.3, 270.9)	0.39 (0.33, 0.46) ***
Non-influenza season	412	463.9	(419.1, 508.7)	208	214.3	(185.2, 243.5)	0.38 (0.31, 0.45) ***
All season	863	971.6	(906.8, 1036.5)	441	454.5	(412.0, 496.9)	0.38 (0.34, 0.44) ***
Age, <65 ^a^							
Influenza season	229	380.8	(331.5, 430.2)	34	110.0	(73.0, 146.9)	0.30 (0.21, 0.43) ***
Non-influenza season	197	327.6	(281.9, 373.4)	53	171.4	(125.3, 217.6)	0.51 (0.37, 0.69) ***
All season	426	708.5	(641.2, 775.8)	87	281.4	(222.3, 340.5)	0.40 (0.31, 0.50) ***
Age, ≥65 ^b^							
Influenza season	222	773.8	(672.0, 875.6)	199	301.0	(259.1, 342.8)	0.41 (0.33, 0.49) ***
Non-influenza season	215	749.4	(649.2, 849.6)	155	234.4	(197.5, 271.3)	0.33 (0.26, 0.40) ***
All season	437	1523.2	(1380.4, 1666.0)	354	535.4	(479.6, 591.1)	0.37 (0.32, 0.42) ***
Female ^c^							
Influenza season	173	389.1	(331.1, 447.1)	83	166.8	(130.9, 202.7)	0.35 (0.26, 0.46) ***
Non-influenza season	166	373.4	(316.6, 430.2)	78	156.8	(122.0, 191.6)	0.34 (0.25, 0.45) ***
All season	339	762.5	(681.3, 843.7)	161	323.6	(273.6, 373.6)	0.34 (0.28, 0.42) ***
Male ^d^							
Influenza season	278	626.7	(553.0, 700.3)	150	317.2	(266.5, 368.0)	0.42 (0.34, 0.52) ***
Non-influenza season	246	554.5	(485.2, 623.8)	130	274.9	(227.7, 322.2)	0.40 (0.32, 0.51) ***
All season	524	1181.2	(1080.1, 1282.4)	280	592.1	(522.8, 661.5)	0.41 (0.35, 0.48) ***

^a^ Total follow-up 60,129.0 person-year for unvaccinated and 30,916.8 for Vaccinated. ^b^ Total follow-up 28,690.2 person-year for unvaccinated and 66,123.2 for Vaccinated. ^c^ Total follow-up 44,458.1 person-year for unvaccinated and 49,754.3 for Vaccinated. ^d^ Total follow-up 44,361.0 person-year for unvaccinated and 47,285.7 for Vaccinated. C.I.: confidence interval. HR: hazard ratio. † Main model is adjusted for age, sex, diabetes, dyslipidemia cerebrovascular diseases, heart diseases, hepatitis B virus, hepatitis C virus, cirrhosis, moderate and severe liver disease, asthma, antihypertensives, diuretics, beta blocking agents, calcium channel blocker, RAA, Statin, Metformin, Aspirin, level of urbanization, monthly income in propensity score. ***: *p* < 0.001.

**Table 3:** Risk of dialysis among Unvaccinated and Vaccinated in Study Cohort.

All Group (*n* = 37,117)	Unvaccinated (Total Follow-Up 89,417.6 Person-Years)			Vaccinated (Total Follow-Up 99,322.0 Person-Years)			Adjusted HR † (95% C.I.)
	No. of Patients with Cancer	Incidence Rate (per 10^5^ Person-Years) (95% C.I.)		No. of Patients with Cancer	Incidence Rate (per 10^5^ Person-Years) (95% C.I.)		
Whole cohort							
Influenza season	159	177.8	(150.2, 205.5)	85	85.6	(67.4, 103.8)	0.40 (0.30, 0.53) ***
Non-influenza season	131	146.5	(121.4, 171.6)	78	78.5	(61.1, 96.0)	0.42 (0.31, 0.57) ***
All season	290	324.3	(287.0, 361.6)	163	164.1	(138.9, 189.3)	0.41 (0.33, 0.51) ***
Age, <65 ^a^							
Influenza season	96	158.6	(126.9, 190.3)	26	82.3	(50.7, 113.9)	0.46 (0.30, 0.72) ***
Non-influenza season	77	127.2	(98.8, 155.6)	19	60.1	(33.1, 87.2)	0.44 (0.26, 0.73) **
All season	173	285.8	(243.2, 328.4)	45	142.5	(100.8, 184.1)	0.45 (0.32, 0.63) ***
Age, ≥65 ^b^							
Influenza season	63	218.1	(164.2, 271.9)	59	87.1	(64.9, 109.3)	0.36 (0.25, 0.52) ***
Non-influenza season	54	186.9	(137.1, 236.8)	59	87.1	(64.9, 109.3)	0.41 (0.28, 0.60) ***
All season	117	405.0	(331.6, 478.4)	118	174.2	(142.8, 205.6)	0.39 (0.30, 0.50) ***
Female ^c^							
Influenza season	77	172.3	(133.8, 210.8)	33	65.2	(43.0, 87.5)	0.30 (0.19, 0.46) ***
Non-influenza season	49	109.6	(78.9, 140.3)	32	63.2	(41.3, 85.2)	0.43 (0.26, 0.69) ***
All season	126	281.9	(232.7, 331.1)	65	128.5	(97.2, 159.7)	0.35 (0.25, 0.48) ***
Male ^d^							
Influenza season	82	183.3	(143.7, 223.0)	52	106.7	(77.7, 135.7)	0.53 (0.36, 0.78) **
Non-influenza season	82	183.3	(143.7, 223.0)	46	94.4	(67.1, 121.7)	0.43 (0.29, 0.63) ***
All season	164	366.7	(310.6, 422.8)	98	201.1	(161.3, 241.0)	0.48 (0.36, 0.63) ***

^a^ Total follow-up 60,526.9 person-year for unvaccinated and 31,588.8 for Vaccinated. ^b^ Total follow-up 28,890.8 person-year for unvaccinated and 67,733.2 for Vaccinated. ^c^ Total follow-up 44,694.3 person-year for unvaccinated and 50,597.0 for Vaccinated. ^d^ Total follow-up 44,723.3 person-year for unvaccinated and 48,725.0 for Vaccinated. C.I.: confidence interval. HR: hazard ratio. † Main model is adjusted for age, sex, diabetes, dyslipidemia cerebrovascular diseases, heart diseases, hepatitis B virus, hepatitis C virus, cirrhosis, moderate and severe liver disease, asthma, antihypertensives, diuretics, beta blocking agents, calcium channel blocker, RAA, Statin, Metformin, Aspirin, level of urbanization, monthly income in propensity score. **: *p* < 0.01 ***: *p* < 0.001.

**Table 4:** Sensitivity Analysis of Adjusted HRs of Vaccination in Risk Reduction of CKD in Influenza Season.

	Unvaccinated	Vaccinated			*p* for Trend
		1	2–3	≥4	
	Adjusted HR (95% C.I.)	Adjusted HR (95% C.I.)	Adjusted HR (95% C.I.)	Adjusted HR (95% C.I.)	
Main model †	1.00	0.59 (0.47, 0.74) ***	0.49 (0.39, 0.60) ***	0.13 (0.09, 0.19) ***	<0.001
Subgroup effects					
Age, years					
<65	1.00	0.40 (0.24, 0.65) ***	0.31 (0.18, 0.55) ***	0.14 (0.05, 0.37) ***	<0.001
≥65	1.00	0.67 (0.52, 0.87) **	0.53 (0.41, 0.67) ***	0.12 (0.08, 0.18) ***	<0.001
Sex					
Female	1.00	0.57 (0.40, 0.82) **	0.40 (0.28, 0.58) ***	0.13 (0.07, 0.23) ***	<0.001
Male	1.00	0.61 (0.46, 0.81) ***	0.54 (0.41, 0.71) ***	0.13 (0.08, 0.21) ***	<0.001
Diabetes					
No	1.00	0.74 (0.54, 1.00)	0.59 (0.44, 0.80) ***	0.14 (0.08, 0.25) ***	<0.001
Yes	1.00	0.47 (0.34, 0.66) ***	0.40 (0.29, 0.55) ***	0.12 (0.07, 0.19) ***	<0.001
Heart diseases					
No	1.00	0.64 (0.44, 0.93) *	0.42 (0.28, 0.64) ***	0.08 (0.03, 0.19) ***	<0.001
Yes	1.00	0.58 (0.44, 0.77) ***	0.52 (0.40, 0.67) ***	0.15 (0.10, 0.23) ***	<0.001
Cerebrovascular diseases					
No	1.00	0.50 (0.37, 0.68) ***	0.43 (0.32, 0.58) ***	0.11 (0.06, 0.18) ***	<0.001
Yes	1.00	0.76 (0.54, 1.07)	0.58 (0.42, 0.81) **	0.16 (0.10, 0.28) ***	<0.001
Asthma					
No	1.00	0.57 (0.44, 0.75) ***	0.50 (0.39, 0.65) ***	0.12 (0.08, 0.19) ***	<0.001
Yes	1.00	0.65 (0.43, 0.99) *	0.44 (0.29, 0.67) ***	0.15 (0.08, 0.28) ***	<0.001
Antihypertensives					
No (<28 days)	1.00	0.61 (0.47, 0.78) ***	0.45 (0.35, 0.58) ***	0.11 (0.07, 0.18) ***	<0.001
Yes (≥28 days)	1.00	0.58 (0.34, 0.96) *	0.65 (0.42, 1.01)	0.18 (0.09, 0.34) ***	<0.001
Diuretics					
No (<28 days)	1.00	0.59 (0.41, 0.85) **	0.45 (0.31, 0.65) ***	0.09 (0.04, 0.19) ***	<0.001
Yes (≥28 days)	1.00	0.61 (0.45, 0.81) ***	0.52 (0.39, 0.68) ***	0.15 (0.10, 0.23) ***	<0.001
Beta blocking agents					
No (<28 days)	1.00	0.50 (0.36, 0.69) ***	0.41 (0.29, 0.56) ***	0.12 (0.07, 0.21) ***	<0.001
Yes (≥28 days)	1.00	0.71 (0.52, 0.97) *	0.57 (0.42, 0.76) ***	0.14 (0.09, 0.23) ***	<0.001
Calcium channel blocker					
No (<28 days)	1.00	0.52 (0.34, 0.80) **	0.45 (0.30, 0.68) ***	0.06 (0.02, 0.17) ***	<0.001
Yes (≥28 days)	1.00	0.63 (0.48, 0.82) ***	0.50 (0.39, 0.65) ***	0.15 (0.10, 0.23) ***	<0.001
RAA					
No (<28 days)	1.00	0.62 (0.43, 0.91) *	0.42 (0.28, 0.63) ***	0.11 (0.05, 0.22) ***	<0.001
Yes (≥28 days)	1.00	0.58 (0.44, 0.77) ***	0.52 (0.40, 0.68) ***	0.14 (0.09, 0.21) ***	<0.001
Statin drugs					
<28 days	1.00	0.61 (0.47, 0.78) ***	0.46 (0.36, 0.59) ***	0.12 (0.08, 0.19) ***	<0.001
28–365 days	1.00	0.61 (0.34, 1.11)	0.74 (0.44, 1.24)	0.15 (0.06, 0.39) ***	<0.001
>365 days	1.00	0.46 (0.20, 1.05)	0.35 (0.16, 0.76) **	0.14 (0.05, 0.40) ***	<0.001
Metformin drug					
<28 days	1.00	0.67 (0.52, 0.87) **	0.50 (0.38, 0.64) ***	0.14 (0.09, 0.22) ***	<0.001
28–365 days	1.00	0.40 (0.19, 0.85) *	0.32 (0.15, 0.70) **	0.09 (0.02, 0.37) ***	<0.001
>365 days	1.00	0.44 (0.24, 0.79) **	0.56 (0.34, 0.91) *	0.10 (0.04, 0.26) ***	<0.001
Aspirin drug					
<28 days	1.00	0.57 (0.43, 0.77) ***	0.47 (0.35, 0.63) ***	0.11 (0.07, 0.19) ***	<0.001
28–365 days	1.00	0.65 (0.42, 1.01)	0.37 (0.23, 0.61) ***	0.12 (0.05, 0.26) ***	<0.001
>365 days	1.00	0.60 (0.33, 1.09)	0.73 (0.45, 1.19)	0.19 (0.09, 0.40) ***	<0.001

*: *p* < 0.05, **: *p* < 0.01, ***: *p* < 0.001. HR: hazard ratio. † Main model is adjusted for age, sex, diabetes, dyslipidemia cerebrovascular diseases, heart diseases, hepatitis B virus, hepatitis C virus, cirrhosis, moderate and severe liver disease, asthma, antihypertensives, diuretics, beta blocking agents, calcium channel blocker, RAA, Statin, Metformin, Aspirin, level of urbanization, monthly income in propensity score.

**Table 5:** Sensitivity Analysis of Adjusted HRs of Vaccination in Risk Reduction of CKD in Non-Influenza Season.

	Unvaccinated	Vaccinated			*p* for Trend
		1	2–3	≥4	
	Adjusted HR (95% C.I.)	Adjusted HR (95% C.I.)	Adjusted HR (95% C.I.)	Adjusted HR (95% C.I.)	
Main model †	1.00	0.66 (0.53, 0.83) ***	0.36 (0.28, 0.47) ***	0.17 (0.12, 0.24) ***	<0.001
Subgroup effects					
Age, years					
<65	1.00	0.74 (0.50, 1.10)	0.36 (0.21, 0.63) ***	0.36 (0.19, 0.69) **	<0.001
≥65	1.00	0.61 (0.47, 0.81) ***	0.35 (0.26, 0.47) ***	0.14 (0.09, 0.20) ***	<0.001
Sex					
Female	1.00	0.74 (0.53, 1.04)	0.21 (0.13, 0.35) ***	0.16 (0.09, 0.28) ***	<0.001
Male	1.00	0.60 (0.45, 0.82) **	0.46 (0.34, 0.63) ***	0.17 (0.11, 0.27) ***	<0.001
Diabetes					
No	1.00	0.72 (0.52, 1.00) *	0.41 (0.28, 0.59) ***	0.11 (0.06, 0.20) ***	<0.001
Yes	1.00	0.62 (0.46, 0.85) **	0.33 (0.23, 0.47) ***	0.23 (0.15, 0.34) ***	<0.001
Heart diseases					
No	1.00	0.56 (0.36, 0.87) **	0.46 (0.30, 0.73) ***	0.13 (0.06, 0.29) ***	<0.001
Yes	1.00	0.71 (0.55, 0.93) *	0.33 (0.24, 0.45) ***	0.18 (0.13, 0.27) ***	<0.001
Cerebrovascular diseases					
No	1.00	0.67 (0.51, 0.89) **	0.32 (0.23, 0.46) ***	0.18 (0.12, 0.28) ***	<0.001
Yes	1.00	0.66 (0.45, 0.96) *	0.42 (0.29, 0.62) ***	0.15 (0.09, 0.27) ***	<0.001
Asthma					
No	1.00	0.65 (0.50, 0.84) **	0.33 (0.24, 0.44) ***	0.17 (0.12, 0.25) ***	<0.001
Yes	1.00	0.74 (0.47, 1.17)	0.48 (0.30, 0.76) **	0.17 (0.09, 0.34) ***	<0.001
Antihypertensives					
No (<28 days)	1.00	0.66 (0.51, 0.87) **	0.35 (0.25, 0.47) ***	0.19 (0.13, 0.29) ***	<0.001
Yes (≥28 days)	1.00	0.67 (0.44, 1.02)	0.40 (0.26, 0.64) ***	0.14 (0.07, 0.26) ***	<0.001
Diuretics					
No (<28 days)	1.00	0.52 (0.35, 0.79) **	0.40 (0.26, 0.60) ***	0.09 (0.04, 0.19) ***	<0.001
Yes (≥28 days)	1.00	0.75 (0.57, 0.99) *	0.35 (0.25, 0.48) ***	0.21 (0.15, 0.31) ***	<0.001
Beta blocking agents					
No (<28 days)	1.00	0.65 (0.47, 0.90) *	0.38 (0.26, 0.56) ***	0.08 (0.04, 0.17) ***	<0.001
Yes (≥28 days)	1.00	0.68 (0.50, 0.93) *	0.35 (0.24, 0.49) ***	0.24 (0.16, 0.35) ***	<0.001
Calcium channel blocker					
No (<28 days)	1.00	0.57 (0.37, 0.87) **	0.25 (0.14, 0.42) ***	0.14 (0.07, 0.28) ***	<0.001
Yes (≥28 days)	1.00	0.71 (0.54, 0.93) *	0.41 (0.31, 0.55) ***	0.18 (0.12, 0.27) ***	<0.001
RAA					
No (<28 days)	1.00	0.64 (0.43, 0.94) *	0.33 (0.21, 0.52) ***	0.10 (0.05, 0.21) ***	<0.001
Yes (≥28 days)	1.00	0.68 (0.52, 0.90) **	0.38 (0.28, 0.52) ***	0.21 (0.14, 0.31) ***	<0.001
Statin drugs					
<28 days	1.00	0.59 (0.44, 0.77) ***	0.35 (0.26, 0.48) ***	0.15 (0.10, 0.23) ***	<0.001
28–365 days	1.00	0.93 (0.58, 1.49)	0.41 (0.23, 0.74) **	0.22 (0.11, 0.45) ***	<0.001
>365 days	1.00	0.81 (0.39, 1.68)	0.37 (0.16, 0.87) *	0.26 (0.10, 0.64) **	0.001
Metformin drug					
<28 days	1.00	0.63 (0.48, 0.84) **	0.39 (0.29, 0.52) ***	0.13 (0.08, 0.21) ***	<0.001
28–365 days	1.00	0.53 (0.28, 0.99) *	0.19 (0.07, 0.47) ***	0.21 (0.08, 0.53) **	<0.001
>365 days	1.00	0.91 (0.55, 1.50)	0.43 (0.24, 0.78) **	0.34 (0.18, 0.64) ***	<0.001
Aspirin drug					
<28 days	1.00	0.60 (0.44, 0.82) **	0.34 (0.23, 0.48) ***	0.13 (0.07, 0.22) ***	<0.001
28–365 days	1.00	0.83 (0.55, 1.27)	0.39 (0.24, 0.64) ***	0.16 (0.08, 0.33) ***	<0.001
>365 days	1.00	0.68 (0.39, 1.19)	0.43 (0.24, 0.75) **	0.29 (0.16, 0.53) ***	<0.001

*: *p* < 0.05, **: *p* < 0.01, ***: *p* < 0.001. HR: hazard ratio. † Main model is adjusted for age, sex, diabetes, dyslipidemia cerebrovascular diseases, heart diseases, hepatitis B virus, hepatitis C virus, cirrhosis, moderate and severe liver disease, asthma, antihypertensives, diuretics, beta blocking agents, calcium channel blocker, RAA, Statin, Metformin, Aspirin, level of urbanization, monthly income in propensity score.

**Table 6:** Sensitivity Analysis of Adjusted HRs of Vaccination in Risk Reduction of CKD in All Season.

	Unvaccinated	Vaccinated			*p* for Trend
		1	2–3	≥4	
	Adjusted HR (95% C.I.)	Adjusted HR (95% C.I.)	Adjusted HR (95% C.I.)	Adjusted HR (95% C.I.)	
Main model †	1.00	0.63 (0.53, 0.74) ***	0.43 (0.36, 0.50) ***	0.15 (0.12, 0.19) ***	<0.001
Subgroup effects					
Age, years					
<65	1.00	0.56 (0.41, 0.76) ***	0.34 (0.23, 0.50) ***	0.25 (0.14, 0.42) ***	<0.001
≥65	1.00	0.64 (0.53, 0.78) ***	0.44 (0.37, 0.53) ***	0.13 (0.10, 0.17) ***	<0.001
Sex					
Female	1.00	0.65 (0.51, 0.84) ***	0.31 (0.23, 0.42) ***	0.14 (0.10, 0.21) ***	<0.001
Male	1.00	0.61 (0.49, 0.75) ***	0.50 (0.41, 0.62) ***	0.15 (0.11, 0.21) ***	<0.001
Diabetes					
No	1.00	0.73 (0.58, 0.91) **	0.50 (0.40, 0.64) ***	0.13 (0.09, 0.19) ***	<0.001
Yes	1.00	0.54 (0.43, 0.68) ***	0.37 (0.29, 0.47) ***	0.17 (0.12, 0.23) ***	<0.001
Heart diseases					
No	1.00	0.60 (0.45, 0.80) ***	0.44 (0.33, 0.60) ***	0.10 (0.05, 0.18) ***	<0.001
Yes	1.00	0.65 (0.53, 0.78) ***	0.42 (0.35, 0.52) ***	0.17 (0.13, 0.22) ***	<0.001
Cerebrovascular diseases					
No	1.00	0.58 (0.48, 0.72) ***	0.38 (0.30, 0.47) ***	0.14 (0.10, 0.20) ***	<0.001
Yes	1.00	0.71 (0.55, 0.92) **	0.51 (0.39, 0.65) ***	0.16 (0.11, 0.23) ***	<0.001
Asthma					
No	1.00	0.61 (0.51, 0.74) ***	0.42 (0.34, 0.51) ***	0.14 (0.11, 0.20) ***	<0.001
Yes	1.00	0.69 (0.51, 0.94) *	0.46 (0.33, 0.62) ***	0.16 (0.10, 0.25) ***	<0.001
Antihypertensives					
No (<28 days)	1.00	0.63 (0.53, 0.76) ***	0.40 (0.33, 0.49) ***	0.15 (0.11, 0.20) ***	<0.001
Yes (≥28 days)	1.00	0.63 (0.46, 0.87) **	0.51 (0.37, 0.70) ***	0.15 (0.10, 0.24) ***	<0.001
Diuretics					
No (<28 days)	1.00	0.56 (0.43, 0.73) ***	0.43 (0.32, 0.56) ***	0.09 (0.05, 0.15) ***	<0.001
Yes (≥28 days)	1.00	0.68 (0.55, 0.82) ***	0.43 (0.35, 0.54) ***	0.18 (0.14, 0.24) ***	<0.001
Beta blocking agents					
No (<28 days)	1.00	0.57 (0.45, 0.72) ***	0.40 (0.31, 0.51) ***	0.10 (0.06, 0.16) ***	<0.001
Yes (≥28 days)	1.00	0.69 (0.56, 0.86) **	0.46 (0.36, 0.57) ***	0.19 (0.14, 0.26) ***	<0.001
Calcium channel blocker					
No (<28 days)	1.00	0.55 (0.40, 0.74) ***	0.35 (0.25, 0.48) ***	0.10 (0.06, 0.18) ***	<0.001
Yes (≥28 days)	1.00	0.67 (0.55, 0.81) ***	0.46 (0.38, 0.56) ***	0.17 (0.13, 0.22) ***	<0.001
RAA					
No (<28 days)	1.00	0.63 (0.48, 0.83) ***	0.38 (0.28, 0.51) ***	0.10 (0.06, 0.17) ***	<0.001
Yes (≥28 days)	1.00	0.63 (0.52, 0.77) ***	0.45 (0.37, 0.55) ***	0.17 (0.13, 0.23) ***	<0.001
Statin drugs					
<28 days	1.00	0.60 (0.49, 0.72) ***	0.41 (0.34, 0.50) ***	0.14 (0.10, 0.18) ***	<0.001
28–365 days	1.00	0.78 (0.54, 1.13)	0.56 (0.38, 0.82) **	0.19 (0.11, 0.33) ***	<0.001
>365 days	1.00	0.62 (0.36, 1.06)	0.36 (0.20, 0.64) ***	0.19 (0.10, 0.38) ***	<0.001
Metformin drug					
<28 days	1.00	0.65 (0.54, 0.79) ***	0.45 (0.37, 0.54) ***	0.14 (0.10, 0.19) ***	<0.001
28–365 days	1.00	0.47 (0.29, 0.76) **	0.25 (0.14, 0.45) ***	0.15 (0.07, 0.33) ***	<0.001
>365 days	1.00	0.65 (0.44, 0.95) *	0.50 (0.34, 0.74) ***	0.21 (0.13, 0.35) ***	<0.001
Aspirin drug					
<28 days	1.00	0.59 (0.47, 0.73) ***	0.41 (0.33, 0.51) ***	0.12 (0.08, 0.18) ***	<0.001
28–365 days	1.00	0.74 (0.55, 1.00) *	0.38 (0.27, 0.54) ***	0.14 (0.08, 0.23) ***	<0.001
>365 days	1.00	0.64 (0.43, 0.97) *	0.57 (0.40, 0.83) **	0.24 (0.15, 0.39) ***	<0.001

*: *p* < 0.05, **: *p* < 0.01, ***: *p* < 0.001. HR: hazard ratio. † Main model is adjusted for age, sex, diabetes, dyslipidemia cerebrovascular diseases, heart diseases, hepatitis B virus, hepatitis C virus, cirrhosis, moderate and severe liver disease, asthma, antihypertensives, diuretics, beta blocking agents, calcium channel blocker, RAA, Statin, Metformin, Aspirin, level of urbanization, monthly income in propensity score.

**Table 7:** Sensitivity Analysis of Adjusted HRs of Vaccination in Risk Reduction of dialysis in Influenza Season.

	Unvaccinated	Vaccinated			*p* for Trend
		1	2–3	≥4	
	Adjusted HR (95% C.I.)	Adjusted HR (95% C.I.)	Adjusted HR (95% C.I.)	Adjusted HR (95% C.I.)	
Main model †	1.00	0.63 (0.44, 0.92) *	0.43 (0.29, 0.63) ***	0.21 (0.13, 0.35) ***	<0.001
Subgroup effects					
Age, years					
<65	1.00	0.90 (0.54, 1.50)	0.15 (0.05, 0.48) **	0.35 (0.14, 0.86) *	<0.001
≥65	1.00	0.47 (0.28, 0.81) **	0.50 (0.32, 0.78) **	0.17 (0.09, 0.31) ***	<0.001
Sex					
Female	1.00	0.52 (0.30, 0.92) *	0.27 (0.14, 0.52) ***	0.15 (0.07, 0.34) ***	<0.001
Male	1.00	0.76 (0.46, 1.25)	0.60 (0.37, 1.00) *	0.29 (0.15, 0.55) ***	<0.001
Diabetes					
No	1.00	0.55 (0.29, 1.06)	0.32 (0.15, 0.65) **	0.28 (0.13, 0.58) ***	<0.001
Yes	1.00	0.68 (0.43, 1.07)	0.50 (0.31, 0.80) **	0.18 (0.09, 0.36) ***	<0.001
Heart diseases					
No	1.00	0.51 (0.25, 1.04)	0.33 (0.15, 0.74) **	0.33 (0.15, 0.74) **	<0.001
Yes	1.00	0.70 (0.45, 1.08)	0.46 (0.29, 0.73) ***	0.17 (0.09, 0.33) ***	<0.001
Cerebrovascular diseases					
No	1.00	0.54 (0.33, 0.88) *	0.30 (0.17, 0.53) ***	0.18 (0.09, 0.36) ***	<0.001
Yes	1.00	0.86 (0.48, 1.52)	0.67 (0.39, 1.18)	0.28 (0.13, 0.61) **	<0.001
Asthma					
No	1.00	0.59 (0.38, 0.91) *	0.44 (0.28, 0.68) ***	0.19 (0.10, 0.34) ***	<0.001
Yes	1.00	0.86 (0.41, 1.82)	0.41 (0.17, 0.98) *	0.31 (0.12, 0.79) *	0.004
Antihypertensives					
No (<28 days)	1.00	0.72 (0.47, 1.09)	0.43 (0.27, 0.68) ***	0.21 (0.12, 0.40) ***	<0.001
Yes (≥28 days)	1.00	0.43 (0.19, 0.98) *	0.42 (0.20, 0.88) *	0.21 (0.09, 0.52) ***	<0.001
Diuretics					
No (<28 days)	1.00	0.71 (0.31, 1.59)	0.67 (0.30, 1.47)	0.17 (0.04, 0.72) *	0.010
Yes (≥28 days)	1.00	0.62 (0.41, 0.94) *	0.38 (0.24, 0.60) ***	0.23 (0.13, 0.39) ***	<0.001
Beta blocking agents					
No (<28 days)	1.00	0.53 (0.30, 0.94) *	0.44 (0.25, 0.77) **	0.16 (0.07, 0.38) ***	<0.001
Yes (≥28 days)	1.00	0.73 (0.45, 1.19)	0.41 (0.24, 0.71) **	0.25 (0.13, 0.46) ***	<0.001
Calcium channel blocker					
No (<28 days)	1.00	0.65 (0.30, 1.40)	0.51 (0.23, 1.11)	0.29 (0.11, 0.75) *	0.004
Yes (≥28 days)	1.00	0.63 (0.41, 0.96) *	0.40 (0.25, 0.63) ***	0.19 (0.11, 0.35) ***	<0.001
RAA					
No (<28 days)	1.00	0.65 (0.32, 1.34)	0.70 (0.37, 1.33)	0.20 (0.07, 0.57) **	0.002
Yes (≥28 days)	1.00	0.62 (0.40, 0.97) *	0.34 (0.20, 0.55) ***	0.22 (0.12, 0.39) ***	<0.001
Statin drugs					
<28 days	1.00	0.54 (0.34, 0.87) *	0.51 (0.33, 0.78) **	0.19 (0.10, 0.35) ***	<0.001
28–365 days	1.00	0.82 (0.38, 1.77)	0.29 (0.10, 0.86) *	0.26 (0.09, 0.79) *	0.003
>365 days	1.00	0.87 (0.30, 2.49)	0.11 (0.01, 0.87) *	0.25 (0.07, 0.93) *	0.008
Metformin drug					
<28 days	1.00	0.57 (0.35, 0.94) *	0.39 (0.23, 0.65) ***	0.25 (0.14, 0.45) ***	<0.001
28–365 days+	1.00	0.47 (0.16, 1.36)	0.16 (0.05, 0.56) **		0.002
>365 days	1.00	0.88 (0.45, 1.73)	0.61 (0.30, 1.23)	0.23 (0.09, 0.62) **	0.002
Aspirin drug					
<28 days	1.00	0.45 (0.25, 0.79) **	0.50 (0.30, 0.82) **	0.18 (0.09, 0.38) ***	<0.001
28–365 days	1.00	0.97 (0.53, 1.79)	0.33 (0.14, 0.75) **	0.13 (0.04, 0.43) ***	<0.001
>365 days	1.00	0.70 (0.28, 1.77)	0.38 (0.14, 1.05)	0.41 (0.17, 1.03)	0.026

*: *p* < 0.05, **: *p* < 0.01, ***: *p* < 0.001. HR: hazard ratio. † Main model is adjusted for age, sex, diabetes, dyslipidemia cerebrovascular diseases, heart diseases, hepatitis B virus, hepatitis C virus, cirrhosis, moderate and severe liver disease, asthma, antihypertensives, diuretics, beta blocking agents, calcium channel blocker, RAA, Statin, Metformin, Aspirin, level of urbanization, monthly income in propensity score.

**Table 8:** Sensitivity Analysis of Adjusted HRs of vaccination in risk reduction of hemodialysis in non-influenza season.

	Unvaccinated	Vaccinated			*p* for Trend
		1	2–3	≥4	
	Adjusted HR (95% C.I.)	Adjusted HR (95% C.I.)	Adjusted HR (95% C.I.)	Adjusted HR (95% C.I.)	
Main model †	1.00	0.90 (0.63, 1.28)	0.42 (0.27, 0.64) ***	0.12 (0.06, 0.24) ***	<0.001
Subgroup effects					
Age, years					
<65	1.00	0.64 (0.33, 1.24)	0.39 (0.17, 0.89) *	0.26 (0.08, 0.84) *	<0.001
≥65	1.00	1.06 (0.68, 1.65)	0.44 (0.26, 0.72) **	0.10 (0.04, 0.22) ***	<0.001
Sex					
Female	1.00	1.23 (0.74, 2.04)	0.23 (0.10, 0.54) ***	0.10 (0.03, 0.32) ***	<0.001
Male	1.00	0.68 (0.41, 1.14)	0.56 (0.34, 0.93) *	0.14 (0.06, 0.33) ***	<0.001
Diabetes					
No	1.00	1.10 (0.58, 2.11)	0.68 (0.34, 1.38)	0.17 (0.05, 0.56) **	0.003
Yes	1.00	0.81 (0.53, 1.24)	0.33 (0.19, 0.57) ***	0.11 (0.05, 0.25) ***	<0.001
Heart diseases					
No	1.00	0.79 (0.42, 1.49)	0.70 (0.37, 1.31)	0.15 (0.05, 0.50) **	<0.001
Yes	1.00	0.94 (0.61, 1.44)	0.30 (0.17, 0.54) ***	0.11 (0.05, 0.25) ***	<0.001
Cerebrovascular diseases					
No	1.00	0.79 (0.48, 1.32)	0.37 (0.19, 0.71) **	0.22 (0.10, 0.48) ***	<0.001
Yes	1.00	1.03 (0.62, 1.71)	0.46 (0.26, 0.83) **	0.05 (0.01, 0.21) ***	<0.001
Asthma					
No	1.00	0.87 (0.57, 1.31)	0.41 (0.25, 0.68) ***	0.15 (0.07, 0.32) ***	<0.001
Yes	1.00	1.03 (0.50, 2.12)	0.45 (0.19, 1.03)	0.05 (0.01, 0.36) **	<0.001
Antihypertensives					
No (<28 days)	1.00	1.01 (0.66, 1.52)	0.40 (0.24, 0.69) ***	0.18 (0.09, 0.38) ***	<0.001
Yes (≥28 days)	1.00	0.70 (0.35, 1.40)	0.46 (0.22, 0.96) *	0.03 (0.01, 0.25) ***	<0.001
Diuretics					
No (<28 days)	1.00	1.22 (0.61, 2.43)	0.63 (0.27, 1.46)	0.26 (0.08, 0.89) *	0.027
Yes (≥28 days)	1.00	0.81 (0.53, 1.22)	0.37 (0.23, 0.61) ***	0.10 (0.04, 0.22) ***	<0.001
Beta blocking agents					
No (<28 days)	1.00	1.17 (0.71, 1.94)	0.49 (0.26, 0.93) *	0.15 (0.05, 0.43) ***	<0.001
Yes (≥28 days)	1.00	0.71 (0.43, 1.17)	0.37 (0.21, 0.65) ***	0.10 (0.04, 0.26) ***	<0.001
Calcium channel blocker					
No (<28 days)	1.00	0.92 (0.41, 2.07)	0.49 (0.20, 1.23)	0.14 (0.03, 0.59) **	0.003
Yes (≥28 days)	1.00	0.89 (0.60, 1.32)	0.40 (0.25, 0.65) ***	0.12 (0.06, 0.26) ***	<0.001
RAA					
No (<28 days)	1.00	1.46 (0.78, 2.77)	0.87 (0.43, 1.77)	0.15 (0.03, 0.62) **	0.016
Yes (≥28 days)	1.00	0.74 (0.48, 1.13)	0.31 (0.18, 0.53) ***	0.12 (0.05, 0.25) ***	<0.001
Statin drugs					
<28 days	1.00	0.80 (0.51, 1.25)	0.41 (0.25, 0.70) ***	0.12 (0.05, 0.29) ***	<0.001
28–365 days+	1.00	0.95 (0.44, 2.07)	0.28 (0.12, 0.69) **		0.006
>365 days	1.00	1.41 (0.58, 3.44)	0.24 (0.05, 1.08)	0.28 (0.08, 1.05)	0.016
Metformin drug					
<28 days	1.00	1.04 (0.65, 1.65)	0.52 (0.30, 0.90) *	0.11 (0.04, 0.31) ***	<0.001
28–365 days	1.00	0.81 (0.36, 1.84)	0.25 (0.07, 0.86) *	0.09 (0.01, 0.65) *	<0.001
>365 days	1.00	0.64 (0.30, 1.36)	0.35 (0.15, 0.82) *	0.16 (0.05, 0.46) ***	<0.001
Aspirin drug					
<28 days	1.00	0.85 (0.51, 1.41)	0.53 (0.30, 0.92) *	0.12 (0.04, 0.33) ***	<0.001
28–365 days	1.00	0.72 (0.38, 1.37)	0.22 (0.08, 0.55) **	0.12 (0.04, 0.40) ***	<0.001
>365 days	1.00	1.58 (0.67, 3.76)	0.55 (0.20, 1.51)	0.13 (0.03, 0.61) **	0.002

*: *p* < 0.05, **: *p* < 0.01, ***: *p* < 0.001. HR: hazard ratio. † Main model is adjusted for age, sex, diabetes, dyslipidemia cerebrovascular diseases, heart diseases, hepatitis B virus, hepatitis C virus, cirrhosis, moderate and severe liver disease, asthma, antihypertensives, diuretics, beta blocking agents, calcium channel blocker, RAA, Statin, Metformin, Aspirin, level of urbanization, monthly income in propensity score.

**Table 9:** Sensitivity Analysis of Adjusted HRs of Vaccination in Risk Reduction of dialysis in all season.

	Unvaccinated	Vaccinated			*p* for Trend
		1	2–3	≥4	
	Adjusted HR (95% C.I.)	Adjusted HR (95% C.I.)	Adjusted HR (95% C.I.)	Adjusted HR (95% C.I.)	
Main model †	1.00	0.75 (0.58, 0.98) *	0.42 (0.32, 0.56) ***	0.17 (0.11, 0.26) ***	<0.001
Subgroup effects					
Age, years					
<65	1.00	0.79 (0.53, 1.18)	0.26 (0.13, 0.50) ***	0.31 (0.15, 0.63) **	<0.001
≥65	1.00	0.74 (0.53, 1.04)	0.47 (0.34, 0.66) ***	0.14 (0.08, 0.22) ***	<0.001
Sex					
Female	1.00	0.80 (0.55, 1.16)	0.26 (0.15, 0.43) ***	0.13 (0.07, 0.25) ***	<0.001
Male	1.00	0.72 (0.51, 1.03)	0.58 (0.41, 0.83) **	0.21 (0.13, 0.35) ***	<0.001
Diabetes					
No	1.00	0.76 (0.48, 1.20)	0.45 (0.28, 0.74) **	0.24 (0.13, 0.45) ***	<0.001
Yes	1.00	0.74 (0.55, 1.02)	0.41 (0.29, 0.59) ***	0.14 (0.08, 0.24) ***	<0.001
Heart diseases					
No	1.00	0.64 (0.40, 1.03)	0.50 (0.31, 0.82) **	0.24 (0.13, 0.47) ***	<0.001
Yes	1.00	0.81 (0.59, 1.10)	0.39 (0.27, 0.55) ***	0.14 (0.09, 0.24) ***	<0.001
Cerebrovascular diseases					
No	1.00	0.64 (0.45, 0.91) *	0.33 (0.22, 0.51) ***	0.20 (0.12, 0.33) ***	<0.001
Yes	1.00	0.95 (0.65, 1.39)	0.56 (0.37, 0.84) **	0.15 (0.08, 0.29) ***	<0.001
Asthma					
No	1.00	0.71 (0.53, 0.96) *	0.42 (0.31, 0.59) ***	0.17 (0.11, 0.27) ***	<0.001
Yes	1.00	0.94 (0.56, 1.59)	0.43 (0.24, 0.78) **	0.17 (0.08, 0.39) ***	<0.001
Antihypertensives					
No (<28 days)	1.00	0.85 (0.63, 1.13)	0.42 (0.30, 0.59) ***	0.20 (0.13, 0.32) ***	<0.001
Yes (≥28 days)	1.00	0.57 (0.34, 0.96) *	0.44 (0.26, 0.74) **	0.12 (0.06, 0.27) ***	<0.001
Diuretics					
No (<28 days)	1.00	0.95 (0.56, 1.60)	0.65 (0.36, 1.15)	0.21 (0.08, 0.54) **	0.001
Yes (≥28 days)	1.00	0.70 (0.52, 0.94) *	0.38 (0.27, 0.53) ***	0.17 (0.11, 0.26) ***	<0.001
Beta blocking agents					
No (<28 days)	1.00	0.79 (0.55, 1.16)	0.46 (0.30, 0.70) ***	0.16 (0.08, 0.31) ***	<0.001
Yes (≥28 days)	1.00	0.72 (0.51, 1.03)	0.39 (0.26, 0.58) ***	0.18 (0.10, 0.29) ***	<0.001
Calcium channel blocker					
No (<28 days)	1.00	0.76 (0.44, 1.32)	0.50 (0.28, 0.91) *	0.22 (0.10, 0.49) ***	<0.001
Yes (≥28 days)	1.00	0.75 (0.56, 1.00)	0.40 (0.29, 0.56) ***	0.16 (0.10, 0.25) ***	<0.001
RAA					
No (<28 days)	1.00	0.99 (0.62, 1.58)	0.77 (0.48, 1.24)	0.18 (0.08, 0.42) ***	<0.001
Yes (≥28 days)	1.00	0.68 (0.50, 0.92) *	0.32 (0.22, 0.46) ***	0.17 (0.11, 0.27) ***	<0.001
Statin drugs					
<28 days	1.00	0.66 (0.47, 0.91) *	0.47 (0.33, 0.65) ***	0.16 (0.10, 0.26) ***	<0.001
28–365 days	1.00	0.88 (0.51, 1.51)	0.43 (0.22, 0.84) *	0.14 (0.05, 0.40) ***	<0.001
>365 days	1.00	1.14 (0.58, 2.25)	0.18 (0.05, 0.59) **	0.27 (0.11, 0.68) **	<0.001
Metformin drug					
<28 days	1.00	0.77 (0.55, 1.08)	0.44 (0.31, 0.65) ***	0.19 (0.12, 0.32) ***	<0.001
28–365 days	1.00	0.65 (0.34, 1.23)	0.28 (0.12, 0.65) **	0.05 (0.01, 0.36) **	<0.001
>365 days	1.00	0.76 (0.46, 1.26)	0.48 (0.28, 0.82) **	0.19 (0.09, 0.40) ***	<0.001
Aspirin drug					
<28 days	1.00	0.62 (0.43, 0.90) *	0.51 (0.35, 0.74) ***	0.16 (0.09, 0.28) ***	<0.001
28–365 days	1.00	0.84 (0.54, 1.31)	0.27 (0.15, 0.50) ***	0.12 (0.05, 0.29) ***	<0.001
>365 days	1.00	1.06 (0.57, 1.97)	0.45 (0.22, 0.91) *	0.28 (0.13, 0.60) **	<0.001

*: *p* < 0.05, **: *p* < 0.01, ***: *p* < 0.001. HR: hazard ratio. † Main model is adjusted for age, sex, diabetes, dyslipidemia cerebrovascular diseases, heart diseases, hepatitis B virus, hepatitis C virus, cirrhosis, moderate and severe liver disease, asthma, antihypertensives, diuretics, beta blocking agents, calcium channel blocker, RAA, Statin, Metformin, Aspirin, level of urbanization, monthly income in propensity score.

## Data Availability

The data supporting the findings of the present research were sourced from NHIRD in Taiwan. Owing to the legal restrictions imposed by the Government of Taiwan related to the Personal Information Protection Act, the database cannot be made publicly available.
